# Clinical and Electroencephalographic Features of the Seizures in Neuronal Surface Antibody-Associated Autoimmune Encephalitis

**DOI:** 10.3389/fneur.2020.00280

**Published:** 2020-05-05

**Authors:** Yan Wang, Yi Yu, Yaping Hu, Ying Li, Fan Song, Ying Wang

**Affiliations:** ^1^Department of Neurology, The First Affiliated Hospital of Dalian Medical University, Dalian, China; ^2^Department of Neurology, The First People's Hospital in Jinzhou, Dalian, China; ^3^Department of General Surgery, The First Affiliated Hospital of Dalian Medical University, Dalian, China

**Keywords:** neuronal surface antibody, autoimmune encephalitis, seizure, LGI1 antibody, NMDAR antibody, GABA_B_R antibody

## Abstract

**Objectives:** To investigate clinical and electroencephalographic features of the seizures in different types of neuronal surface antibody (NSAb)-associated autoimmune encephalitis (AE).

**Methods:** The clinical data of the seizures were analyzed in 18 patients with NSAb-associated AEs diagnosed in the First Affiliated Hospital of Dalian Medical University.

**Results:** From May 2013 to April 2019, a total of 18 cases of NSAb-associated AE were diagnosed, including 9 cases of leucine-rich glioma-inactivated 1 protein (LGI1) antibody-associated encephalitis, 7 cases of anti-*N*-methyl-d-aspartate receptor (NMDAR) encephalitis, and 2 cases of anti-γ-aminobutyric acid B receptor (GABA_B_R) encephalitis. All nine cases (100%) with LGI1 AE had seizures manifesting in three types: faciobranchial dystonia seizure (FBDS) (44.4%), mesial temporal lobe epilepsy (MTLE)-like seizure (66.7%), and focal to bilateral tonic–clonic seizure (FBTCS) (77.8%). Six of nine (66.7%) showed abnormal signal on hippocampus or basal ganglia in brain MRI. Five of seven cases (71%) with anti-NMDAR encephalitis had seizures manifesting in three types: focal aware seizure (40%), focal-impaired awareness seizure (20%), generalized tonic–clonic seizure (GTCS) (100%), and status epilepticus (SE) (40%). Three of seven (42.8%) showed abnormalities in brain MRI. Both patients with anti-GABA_B_R encephalitis had seizures manifesting in two types: GTCS and MTLE-like seizure, one with SE. One showed abnormal signal on left hippocampus in brain MRI. All patients (100%) with three types of AE had abnormalities in electroencephalogram (EEG), showing diffuse (4/18) or focal slow waves (14/18) in background, interictal (10/18), or ictal (6/18) epileptic discharges in the temporal or other regions; two patients with anti-NMDAR encephalitis showed delta activity or rhythm in frontotemporal region. All patients with seizures showed good response to immunotherapy except one with LGI1 AE.

**Conclusions:** Most patients with NSAb-associated AE had seizures; seizure types varied between different types of AE. In LGI1 AE, the hippocampus and basal ganglia were two main targets; the corresponding seizure type was MTLE-like seizure and FBDS, respectively. Anti-NMDAR encephalitis had more generalized than focal seizures. Delta activity or rhythm in the frontotemporal region in EEG was helpful for diagnosis. Anti-GABA_B_R encephalitis was characterized by refractory seizures as initial symptom, mainly GTCS or MTLE-like seizure. Most seizures in NSAb-associated AE showed good response to immunotherapy, and antiepileptic drugs should be considered as an add-on symptomatic treatment.

## Introduction

Autoimmune encephalitis (AE) is a new category of encephalitis mediated by autoimmune mechanisms (1). According to different antigens targeted by immune responses, autoimmune encephalitis can be divided into the following types: intraneuronal antibody-associated AE (paraneoplastic limbic encephalitis), neuronal surface antibody (NSAb)-associated AE, intracellular synaptic protein antibody-associated encephalitis, which is between the two types mentioned above such as glutamate decarboxylase (GAD) antibody-associated encephalitis, and other autoimmune encephalitis without definite antigens such as acute disseminated encephalomyelitis (ADEM) (2, 3). Compared with paraneoplastic limbic encephalitis, NSAb-associated encephalitis is a novel category of autoimmune encephalitis with antibodies targeting neuronal surface antigens (such as ion channels, receptors, proteins, etc.), mediating relatively reversible neuronal dysfunction mainly through humoral immune mechanism, and the effect of immunotherapy is good. The paraneoplastic limbic encephalitis, whose antibodies are against intraneuronal antigen, can lead to irreversible neuronal damage through T-cell-mediated immune response, and the effect of immunotherapy is poor (2, 3). Since anti-*N*-methyl-d-aspartate receptor (NMDAR) encephalitis was described in 2007 (4), a series of antibodies against the neuronal surface antigens have been found, such as leucine-rich glioma-inactivated 1 protein (LGI1) antibody, contactin-associated protein-like 2 (Caspr2) antibody, α-amino-3-hydroxy-5-methyl-4-isoxazole-propionic acid receptor (AMPAR) antibody, γ-aminobutyric acid B receptor (GABA_B_R) antibody, glycine receptor (GlyR) antibody, and so on. The AE mediated by these antibodies accounted for 10–20% of all types of encephalitis, and the most common subtype among them is anti-NMDAR encephalitis, which accounts for ~80% of AE, followed by LGI1 antibody-associated encephalitis (LGI1 AE) and anti-GABA_B_R encephalitis (2). There are similar clinical characteristics between different subtypes of NSAb-associated AE with different antibodies, but each subtype has its own features in clinical manifestation, imaging, electroencephalogram (EEG), and incidence of tumor. Most patients with NSAb-associated AE have epileptic seizures in the acute or subacute phase of the disease or later during disease progression (5–8), but little is known about the clinical and EEG features, treatment and prognosis of these seizures, as well as the risk of developing chronic epilepsy. In the present study, 18 cases with NSAb-associated AEs diagnosed in the First Affiliated Hospital of Dalian Medical University were collected and analyzed to investigate the clinical and EEG features of the seizures in different types of NSAb-associated AEs.

## Materials and Methods

### Patients

Eighteen patients diagnosed with NSAb-associated AEs in the Neurology Department of the First Affiliated Hospital of Dalian Medical University between May 2013 and April 2019 were enrolled, who met the following inclusion criteria (1): (1) acute or subacute onset (rapid progression of <3 months) of epileptic seizures, memory impairment, and mental and behavioral abnormalities; (2) seizure occurrence in acute or subacute phase of the disease; (3) positive results of NMDAR, GABA_B_R, LGI1, or Caspr2 antibody in cerebrospinal fluid (CSF) and serum; and (4) immunotherapy as initial treatment, including corticosteroids, immunoglobulin, or a combination of both.

This study was approved by the Medical Ethics Committee of the First Affiliated Hospital of Dalian Medical University, Dalian, China. Written informed consents were obtained from all the patients enrolled in this study.

### Clinical Information

The clinical data of 18 patients of NSAb-associated AEs were collected and analyzed by the authors through electronic medical records, including demographic information, clinical manifestations, EEG, imaging, as well as treatment and outcome. All the patients in our study received a 1.5-/3.0-T MRI scan and a 2-h video EEG (vEEG) recording using the 10–20 system of scalp electrode placement. The antibodies, including NMDAR, GABA_B_R, LGI1, and Caspr2, were detected in the patients' cerebrospinal fluid (CSF) and serum samples. All patients were screened for systemic tumors by imaging and tumor markers. Seizure classification was based on clinical symptoms and EEG as evaluated by neurologists according to the International League Against Epilepsy (ILAE) 2017 classification proposal (9). Status epilepticus (SE) was defined as prolonged seizure activity or recurrent seizures without full recovery of consciousness between episodes for more than 5 min (10). Patients' seizure outcome and additional therapy were followed up every 3 months by telephonic interview and/or clinic visits after discharges.

### Statistical Analysis

Statistical analysis was performed using SPSS for Windows (version 19.0). Descriptive statistics were performed for each variable including means, medians, and standard deviations.

## Results

From May 2013 to April 2019, a total of 18 cases of NSAb-associated AEs were diagnosed in our hospital, including 9 cases of LGI1 AE, 7 cases of anti-NMDAR encephalitis, and 2 cases of anti-GABA_B_R encephalitis. In this group of 18 patients, 10 were male and 8 were female; the ratio of male to female was 10:8. The average age was 46 ± 16 years old (21-67). The course of disease ranged from 9 days to 13 years, and the median duration was 2 months.

### The Clinical Features of Seizures in Nine Cases of LGI1 AE

Table 1 shows clinical features of seizures in nine cases of LGI1 AE. In this group, four were male and five were female; the gender ratio was 4:5. The average age was 52 ± 14 years old (21–67 years old). The course of disease ranged from 9 days to 3 years, and the median duration was 90 days. All nine patients had seizures; the incidence rate was 100%. The seizures manifested in three types: faciobranchial dystonia seizure (FBDS) in four patients (44.4%), mesial temporal lobe epilepsy (MTLE)-like seizure in six patients (66.7%), and focal to bilateral tonic–clonic seizures (FBTCS) in seven patients (77.8%). Subclinical seizures were observed in three patients (33.3%). FBDS manifested as frequent, transient dystonia-like movements in the unilateral face and upper limb, and sometimes, the lower limb was also involved, with consciousness preserved, which continued for a few seconds and a lot of times per day. The symptom of MTLE-like seizure was similar to that of classic MTLE, manifested as paroxysmal consciousness loss with staring and automatisms, which lasted for 1 or 2 min.

**Table 1:** Clinical features of seizures in 9 cases with leucine-rich glioma inactivated 1 protein (LGI1) antibody associated autoimmune encephalitis (AE).

**Case**	**Seizure type**	**Brain MRI**	**vEEG**	**LGI 1 Ab titer in CSF/serum**	**Treatment**	**Outcome**
1	MTLE-like seizure; FBTCS; subclinical seizure	Normal	**Background**: bilateral frontotemporal slow waves; **Interictal**: bilateral sphenoidal electrodes spikes; **Ictal**: MTLE-like seizure	1:10/1:100	Corticoteroids+LEV	Improved
2	MTLE-like seizure; FBTCS	Hyperintensity in left hippocampus	**Background**: diffuse slow waves; **Interictal**: bilateral temporal spikes	1:32/1:320	Corticoteroids+LEV	Improved
3	MTLE-like seizure; FBTCS; FBDS	Hyperintensity in right hippocampus	**Background**: right occipital, parietal, posterior temporal slow waves	1:3.2/1:100	Corticoteroids+IVIG+LEV	Improved
4	MTLE-like seizure; subclinical seizure	Hyperintensity in both hippocampus	**Background**: bilateral frontotemporal slow waves; **Interictal**: bilateral temporal spikes; **Ictal**: MTLE-like seizure	1:1/1:32	Corticoteroids+IVIG+LEV	Improved
5	FBTCS; FBDS	Hyperintensity in right basal ganglia	**Background**: focal slow waves; **Ictal**: FBDS	1:3.2/1:100	CBZ	Improved
6	FBTCS; FAS; FBDS	Normal	**Interictal**: bilateral frontotemporal sharps	1:1/1:10	Corticoteroids+LEV	Improved
7	MTLE-like seizure; FBTCS	Hyperintensity in both hippocampus	**Background**: right occipital, parietal, posterior temporal slow waves; **Ictal**: MTLE-like seizure	1:1/1:10	Corticoteroids+IVIG+LEV	Improved
8	MTLE-like seizure; subclinical seizure	Normal	**Interictal**: bilateral temporal spikes	1:1/1:32	Corticoteroids+OXC	Improved
9	FBTCS; FBDS	Hyperintensity in left basal ganglia	**Background**: bilateral frontotemporal slow waves; **Interictal**: bilateral temporal spikes; **Ictal**: FBDS	1:10/1:100	Corticoteroids+IVIG+multiple AEDs	Improved

*AEDs, antiepileptic drugs; CBZ, carbamazepine; CSF, cerebrospinal fluid; FAS, facal aware seizures; FBDS, faciobranchial dystonia seizure; FBTCS, focal to bilateral tonic–clonic seizures; IVIG, intravenous immunoglobulin; LEV, levetiracetam; MTLE, mesial temporal lobe epilepsy; OXC, oxcarbazepine; d, day; m, month; y, year*.

**Figure 1:**
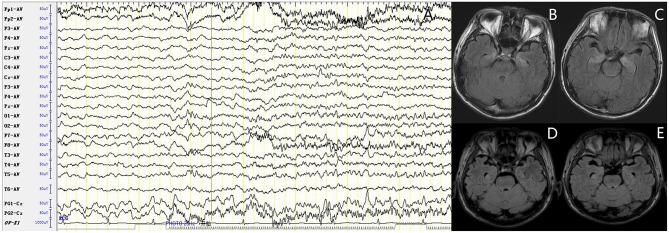
Case 4, male, 62 years old, who was diagnosed with leucine-rich glioma-inactivated 1 protein (LGI1) antibody associated autoimmune encephalitis (AE). **(A)** Ictal electroencephalogram (EEG) of a subclinical seizure showed rhythmic sharp wave discharges in bilateral temporal regions, with amplitude and frequency modulated gradually. **(B,C)** Twenty-three days after onset and before immunotherapy, brain MRI showed high T2/fluid-attenuated inversion recovery (FLAIR) signal on bilateral hippocampus. **(D,E)** About 3 months after onset and after immunotherapy, brain MRI showed significant improvement in the high T2/FLAIR signal on bilateral hippocampus.

**Figure 2:**
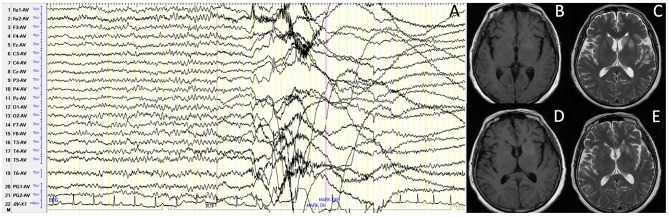
Case 9, female, 63 years old, who was diagnosed with leucine-rich glioma-inactivated 1 protein (LGI1) antibody-associated autoimmune encephalitis (AE). **(A)** Ictal electroencephalogram (EEG) of faciobranchial dystonia seizure (FBDS) showed that 1 s before the clinical onset, the amplitude of all the leads suppressed, followed by artifact of movements, which continued for 5 s, and then recovered to background. **(B,C)** Nineteen days after onset and before immunotherapy, brain MRI showed high T1/T2 signal on the left basal ganglia (caudal nucleus and lenticular nucleus). **(D,E)** Thirty-three days after onset (10 days after immunotherapy), brain MRI showed significant improvement in the high T1/T2 signal on the left basal ganglia.

For accessory examinations, all nine patients underwent brain MRI, and six (66.7%) were abnormal. Among them, two patients showed high-intensity signal in T2/fluid-attenuated inversion recovery (FLAIR)-weighed images on bilateral hippocampus, two patients showed high-intensity signal in T2/FLAIR on unilateral hippocampus (Figures 1B–E), and two patients showed high-intensity signal in T1/T2 on unilateral basal ganglia (caudal nucleus and lenticular nucleus) (Figures 2B–E). The abnormal signals in the brain MRI of the above six cases significantly improved or disappeared after immunotherapy. All nine patients received 2-h-long vEEG monitoring, and all of them (100%) showed abnormalities. Among them, one patient (11.1%) showed diffuse slow waves, eight patients (88.9%) showed focal slow waves in background activities, six patients (66.7%) revealed interictal epileptic discharges such as spikes or sharps in unilateral or bilateral temporal or other brain regions, ictal EEG were recorded in five patients, two were FBDS, and three were MTLE-like seizure. Three patients (33.3%) had subclinical electrographic seizures that originated from the mesial temporal lobe. The clinical manifestation of MTLE-like seizure was paroxysmal sputum with automatisms, which lasted for 1 or 2 min, and ictal EEG showed focal seizure originating from the unilateral temporal region (Figure 1A). The clinical manifestations of FBDS were frequent, brief dystonia-like movements in the unilateral face, upper limb, and even lower limb, with consciousness preserved and relieved within a few seconds; ictal EEG showed 1 s before the clinical onset that the amplitude of all the leads suppressed, followed by artifact of movements, which continued for a few seconds and then recovered to background (Figure 2A).

For the treatment, four of nine patients were treated with corticosteroids combined intravenous immunoglobulin (IVIG) and antiepileptic drugs (AEDs), four patients were treated with corticosteroids and AEDs, and one patient was only treated with AED without immunotherapy. All nine patients were treated with AED; among them were six with levetiracetam (LEV), one with carbamazepine (CBZ), one with oxcarbazepine (OXC), and one with multiple AEDs [LEV, CBZ, topiramate (TPM), and valprate (VPA)] due to intractable FBDS. Eight patients (8/9) were treated with AED for associated seizures before diagnosis and immunotherapy but with poor effect. Eight patients (8/9) improved significantly, who were seizure free after immunotherapy; only one patient still had FBDS after being treated with corticosteroids, IVIG, and multiple AEDs.

We performed a continuous follow-up on all nine patients after discharge, with two of whom were lost. None of the remaining seven patients developed a recurrence during the follow-up period (10–45 months). Four of the seven patients achieved clinical remission and were seizure free, who stopped oral corticosteroids and AED at 6 months after immunotherapy; two patients continued oral corticosteroids and AED due to uncontrolled FBDS and interictal epileptiform discharge in EEG, respectively, and one patient continued oral corticosteroids due to remained memory impairment.

### The Clinical Features of Seizures in Seven Cases of Anti-NMDAR Encephalitis

Table 2 shows clinical features of seizures in seven cases of anti-NMDAR encephalitis. In this group, four were male and three were female; the gender ratio was 4:3. The average age was 34 ± 14 years old (21–63 years old). The course of disease ranged from 9 days to 13 years, and the median duration was 2 months. Five of seven cases had seizures; the incidence rate was 71%. The seizures manifested in three forms: focal aware seizure (FAS) in two patients (40%), focal impaired awareness seizure (FIAS) in one case (20%), generalized tonic–clonic seizure (GTCS) in five cases (100%), and status epilepticus (SE) in two cases (40%).

**Table 2:** Clinical features of seizures in 7 cases with anti-N-methyl-D-aspartate receptor (NMDAR) encephalitis.

**Case**	**Seizure type**	**Brain MRI**	**vEEG**	**NMDAR Ab titer in CSF/serum**	**Treatment**	**Outcome**
10	GTCS; FIAS; SE	Normal	**Background**: diffuse slow waves; delta activity or rhythm in the frontal region	1:1/1:10	Corticoteroids+IVIG+LEV	Improved
11	GTCS; SE	Normal	**Background:** left temporal slow waves; **Interictal**: left posterior temporal spikes	1:3.2/1:10	Corticoteroids+IVIG+CBZ	Improved
12	GTCS	Hyperintensity in right hippocampus	**Background**: right frontotemporal slow waves; **Interictal**: right frontotemporal sharps	1:32/1:320	Corticoteroids+VPA	Improved
13	GTCS; FAS	Hyperintensity in right frontal region	**Background**: diffuse slow waves; **Interictal**: right frontotemporal sharps	1:10/1:100	Corticoteroids+IVIG+LEV	Improved
14	GTCS; FAS	Normal	**Background**: delta activity or rhythm in the frontal region	1:10/1:320	Corticoteroids+(LEV+TPM)	Improved
15	_	Hyperintensity in left parietal region	**Background**: diffuse slow waves	1:1/1:10	Corticoteroids	Improved
16	_	Normal	**Background**: focal slow waves	1:1/1:32	Corticoteroids+IVIG	Improved

*CBZ, carbamazepine; CSF, cerebrospinal fluid; FAS, facal aware seizures; FIAS, facal-impaired awareness seizures; GTCS, generalized tonic-clonic seizure; IVIG, intravenous immunoglobulin; LEV, levetiracetam; SE, Status epilepticus; TPM, topiramete; VPA, Valproic acid; d, day; m, month; y, year*.

**Figure 3:**
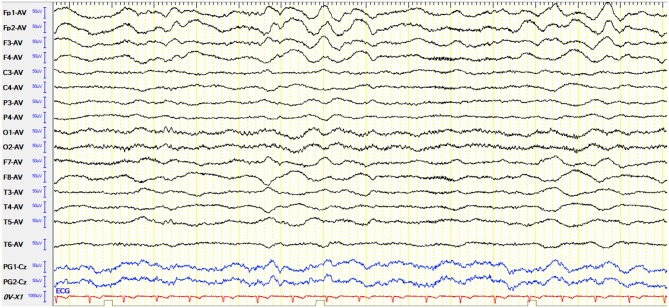
Case 10, male, 25 years old, who was diagnosed with anti-*N*-methyl-d-aspartate receptor (NMDAR) encephalitis. Electroencephalogram (EEG) showed delta activity or rhythm in bilateral frontotemporal region.

For accessory examinations, all seven patients underwent brain MRI, and three (42.8%) showed abnormalities. Among them, one patient showed high T2/FLAIR signal in the right hippocampus, two patients showed high T2/FLAIR signal in the focal regions of the neocortex (one in the frontal lobe, the other in the parietal lobe). All seven cases (100%) had abnormalities in EEG; among them, two cases (28.5%) showed delta activity or rhythm in the frontotemporal region (Figure 3), three cases (42.8%) showed diffuse slow waves, four cases (57.1%) showed focal slow wave activities in background, three cases (42.8%) showed interictal epileptic discharges, and no ictal phase was detected in all the patients.

For treatment, five patients (5/7) were treated with corticosteroids, IVIG, and AEDs; two patients without seizures were treated with corticosteroids with or without IVIG. The five patients with seizures were all treated with AEDs; among them, one was with LEV and TPM, two with LEV, one with VPA, and one with CBZ. All seven patients improved significantly after immunotherapy and were seizure free in the five patients who had seizures.

We performed a continuous follow-up (7–32 months) on all seven patients after discharge. Among them, one patient who achieved clinical remission and ceased oral corticosteroids and AED at 9 months developed a recurrence at 25 months and improved after treated with corticosteroids, IVIG, mycophenolate mofetil, and AEDs. At 6 months after discharge, four patients achieved clinical remission and were seizure free and stopped oral corticosteroids and AED; one patient continued a small dosage of oral corticosteroids and AED due to positive antibodies in the serum, and one patient who had discontinued AED received mycophenolate mofetil combined with a small dosage of oral corticosteroids due to positive antibodies in the cerebrospinal fluid. These two patients mentioned above stopped all medications at 8 and 19 months, respectively.

### The Clinical Features of Seizures in Two Cases of Anti-GABA_B_R Encephalitis

Table 3 shows clinical features of seizures in two cases of anti-GABA_B_R encephalitis. In this group, both were male, 64 and 59 years old, respectively. The course of disease was 15 days and 4 months, respectively. Both of them had seizures, which manifested in two types: GTCS in two patients, MTLE-like seizure in one patient, status epilepticus in one patient, and AEDs were ineffective.

**Table 3:** Clinical features of seizures in 2 cases with anti- γ-aminobutyric acid B receptor (GABA_B_R) encephalitis.

**Case**	**Seizure type**	**Brain MRI**	**vEEG**	**GABA_B_R Ab titer in CSF/ serum**	**Treatment**	**Outcome**
17	GTCS; MTLE-like seizure; SE	Hyperintensity in left hippocampus	**Background**: left temporal slow waves; **Ictal**: a subclinical seizure originating from left temporal region	1:10/1:100	Corticoteroids+IVIG+(LEV+VPA)	Improved
18	GTCS	Normal	**Background**: focal slow waves; **Interictal:** left temporal atypical sharps	1:3.2/1:32	Corticoteroids+CBZ	Improved

*CBZ, carbamazepine; CSF, cerebrospinal fluid; GTCS, generalized tonic-clonic seizure; IVIG, intravenous immunoglobulin; LEV, levetiracetam; MTLE, mesial temporal lobe epilepsy; SE, Status epilepticus; VPA, valproic acid; d, day; m, month*.

**Figure 4:**
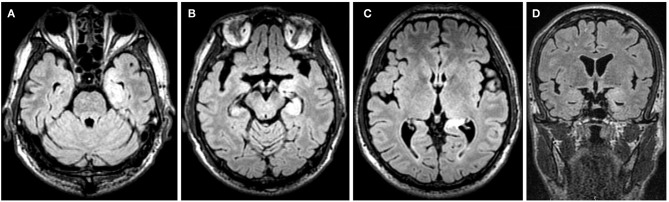
Case 17, male, 64 years old, who was diagnosed with anti-γ-aminobutyric acid B receptor (GABA_B_R) encephalitis. **(A–D)** Twenty-one days after onset and before immunotherapy, brain MRI showed high T2/fluid-attenuated inversion recovery (FLAIR) signal in the left hippocampus.

**Figure 5:**
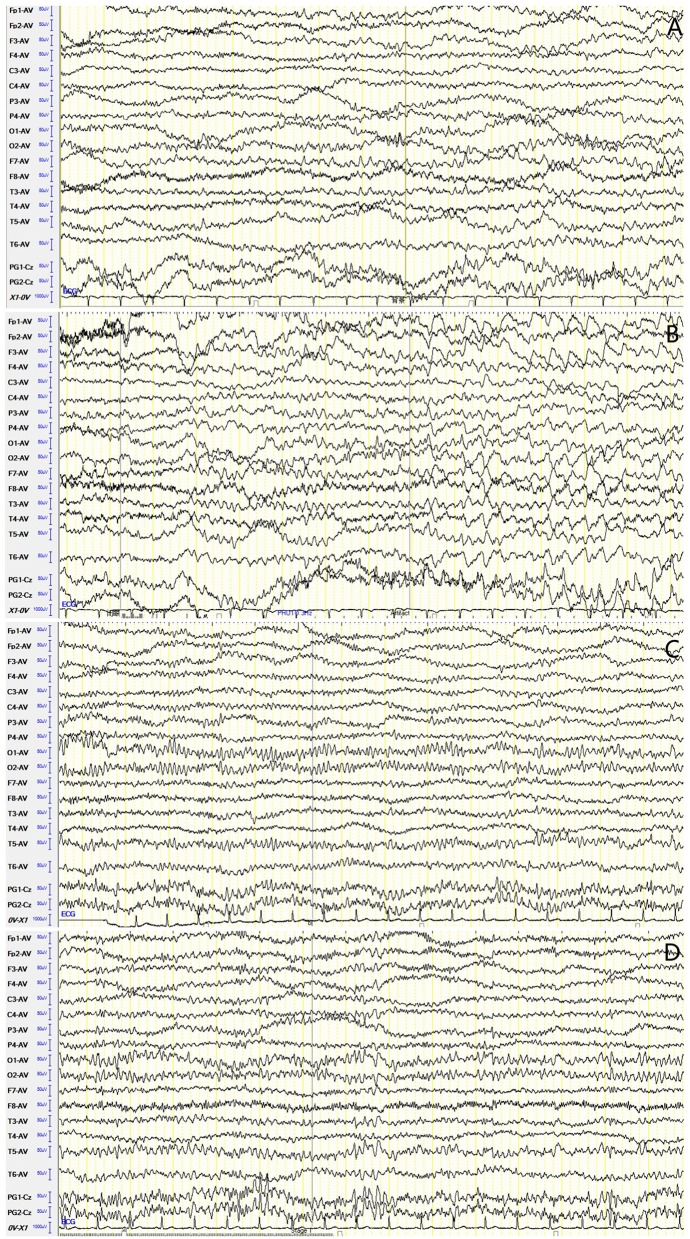
Case 17, male, 64 years old, who was diagnosed with anti-γ-aminobutyric acid B receptor (GABA_B_R) encephalitis. **(A,B)** Twenty-one days after onset and before immunotherapy, electroencephalogram (EEG) showed slow waves in the left temporal region **(A)**, and a subclinical electrographic seizure originating from the left temporal region **(B)**. **(C,D)** Ten days after immunotherapy, EEG showed significant improvement in background activities **(C)**, with scatted slow waves in the left posterior region **(D)**.

For accessory examinations, both patients underwent brain MRI, and one had abnormalities, which showed high T2/FLAIR signal in the left hippocampus (Figures 4A–D). Both patients had abnormalities in EEG; among them, one case showed slow wave activities in the left temporal region (Figure 5A) and a subclinical electrographic seizure originating from the left temporal region (Figure 5B); the other showed atypical sharps in the left temporal region.

For the treatment, one patient was treated with corticosteroids combined with IVIG and AEDs (LEV and VPA), who improved significantly and was seizure free, and EEG recorded 10 days after immunotherapy showed significant improvement in background activities with scattered focal slow waves in the left posterior region (Figures 5C,D). The other was treated with corticosteroids and AED (CBZ), who also improved significantly and was seizure free.

Both patients received continuous follow-up after their discharge. No recurrence was found in any of them during the follow-up period (6–13 months). One patient died of small cell lung carcinoma (SCLC) at 6 months after discharge. The other who achieved clinical remission and was seizure free discontinued oral corticosteroids and AED at 6 months, and still in follow-up.

## Discussion

Most patients with NSAb-associated AE have seizures, particularly LGI1 AE, anti-NMDAR encephalitis, and anti-GABA_B_R encephalitis. However, the incidence rate, seizure type, EEG, treatment and prognosis of these seizures, as well as the risk of developing chronic epilepsy are different between different types of AE. For the first time, we investigated and compared the clinical and EEG features of seizures in three common types of AE, respectively. We found that there was a trend that the incidence rate of seizures was higher in LGI1 AE and anti-GABA_B_R encephalitis than in anti-NMDAR encephalitis. The common seizure types of LGI1 AE were FBDS, MTLE-like seizure, and FBTCS; for anti-GABA_B_R encephalitis, they were GTCS and MTLE-like seizure, which were the initial and main symptoms and often developed SE; while anti-NMDAR encephalitis had various seizure types, more generalized than focal seizures. EEG changes in three types of AE were non-specific, showing diffuse or focal slow waves in background activities, interictal epileptic discharges in the temporal lobe or other regions, and ictal EEG usually originating from the medial temporal region. The positive abnormal rate of ictal EEG in FBDS was low. Besides extreme delta brush, delta activity or rhythm in the frontotemporal region was also a specific EEG feature in anti-NMDAR encephalitis. Most seizures in these three types of AE showed good response to immunotherapy; AEDs should be considered as an add-on symptomatic treatment.

### The Clinical and EEG Features of Seizures in LGI1 AE

In our study, all nine cases with LGI1 AE had seizures; the incidence rate was 100%. The seizures manifested in three types: FBDS (44.4%), MTLE-like seizure (66.7%), and FBTCS (77.8%). These results were consistent with previous studies (5, 6, 11–15), which showed that 90% of the patients with LGI1 AE had seizures that mainly present in three types: FBDS, MTLE-like seizure, and FBTCS (11–14). In a recent study, patients with LGI1 AE were divided into the following three groups based on seizure semiology: FBDS only (FBDS-only), epileptic seizure without FBDS (non-FBDS, mainly manifested MTLE-like semiology), and coexistence of FBDS and other seizure (FBDS+) (16). FBDS is the most specific seizure type in LGI1 AE, which presents in approximately half of the patients (11–13, 17, 18). It is characterized as frequent (>100 times/day), brief (<3 s) dystonia-like movements in the unilateral face, upper limb, and even lower limb with consciousness preserved. FBDS is usually easy to identify and diagnose, but only a few EEG recordings show changes during the ictal phase (15, 17, 18). Minor improvements are found after treatment with AEDs, but immunotherapy is effective. However, to date, the origin of FBDS remains controversial. Cortical, subcortical, and cortical–subcortical origins have been supported by different studies, respectively (3, 16, 17, 19, 20). In the present study, the ictal EEG of FBDS showed suppression of amplitude in all the electrodes 1 s before onset, indicating that it was epileptic in nature. Combined with imaging findings (brain MRI showed high T1/T2 signal on the unilateral basal ganglia), the origin of FBDS was considered subcortical basal ganglia. Previous studies have found that 66–89% of patients with LGI1 AE have MTLE-like seizure (3, 11, 13, 16, 21, 22), which are characterized by fear, epigastric rising, staring, and automatisms. The frequency of this kind of seizure is more frequent than that of classic MTLE, and the duration is relatively shorter (16), which are consistent with our study. The differences in seizure semiology between LGI1 AE and classic MTLE may be attributed to different pathogenic mechanisms (16, 23, 24). Previous reports showed that more than 60% of patients have GTCS, usually in the late stage of the disease (11, 13, 17, 22). In our study, 77.8% of the patients had GTCS. Based on the seizure type, in combination with imaging and EEG in our study, it can be concluded that LGI1 AE commonly affected two locations: one was the limbic system (the medial temporal lobe, hippocampus, and amygdala), of which the seizure type at this time was MTLE-like seizure; the other was the basal ganglia, of which the seizure type was FBDS. These results were slightly different from a previous report, which showed that the motor cortex and hippocampus may be two main targets in LGI1 AE (3, 15, 22).

In our study, 100% of the patients showed abnormalities in EEG; among them, 11.1% showed diffuse slow waves, 88.9% showed focal wave activities, and 66.7% revealed interictal epileptic discharges in the temporal or other brain regions. Ictal EEG were recorded in five patients (55.6%); two were FBDS, and three were MTLE-like seizure. These data indicated that no specific changes were found in EEG of patients with LGI1 AE, with focal or diffuse slow waves in background activities, or interictal epileptic discharges, which was similar to previous studies (6, 15, 25). Ictal EEG of FBDS could not show the origin of epileptic discharge, while ictal EEG of MTLE-like seizure showed epileptic discharge originating from the unilateral medial temporal region. The low positive rate of ictal EEG in FBDS was consistent with previous studies, which revealed that the abnormal rate of EEG for FBDS was only 13–40% and may be due to a deeply located or highly localized epileptogenic zone (15, 17).

In our study, eight patients (88.9%) received immunotherapy and AEDs, and one patient (11.1%) received AED without immunotherapy. Eight patients (88.9%) were treated with AED for seizures before immunotherapy but with poor effect. These patients improved significantly and were seizure free after immunotherapy except for one. These data indicated good effect of immunotherapy, and AEDs should be considered as an add-on symptomatic treatment, which was in line with previous reports (3, 5, 13, 15, 26). Previous studies showed that immunotherapy was effective in 80% of patients with LGI1 AE (13). Seizures, particularly FBDS, disappear immediately after immunotherapy (11, 12). For seizures, broad-spectrum AEDs such as LEV, VPA, TPM, and benzodiazepines were suitable; sodium-channel blockers such as oxcarbazepine and carbamazepine were not recommended because of high incidence of specific cutaneous adverse reaction and hyponatremia (already present in a subset of patients due to a concomitant syndrome of inappropriate antidiuretic hormone secretion) in patients with LGI1 AE (2). However, a more recent study showed that CBZ was more effective than LEV in reducing seizures in LGI1 AE (5), and LEV may lead to psychiatric and behavioral adverse events (3, 5, 27), so the appropriate AEDs for seizures in LGI1 AE need further investigation. In our study, the AED used in six cases was LEV; one was treated with OXC, one with CBZ, and one with multiple AEDs due to intractable FBDS, which could not tell the preferable antiepileptic drug for the seizures in LGI1 AE. Despite the difficulties in treatment, the long-term outcome of the seizures in patients with LGI1AE is good (3, 27). The risk of developing to chronic epilepsy was 15% (13), so long-term AED treatment may be unnecessary (3, 5, 6, 13). The recurrence rate of the disease was between 27 and 35%, suggesting that long-term immunotherapy is necessary (21, 28). However, we do not have sufficient statistical data about the recurrence rate and risk of developing to chronic epilepsy due to short follow-up period and small sample size of our study.

### The Clinical and EEG Features of Seizures in Anti-NMDAR Encephalitis

Epileptic seizure is a common presentation of anti-NMDAR encephalitis (5, 6, 13, 29, 30). In our study, 71% of the patients with anti-NMDAR encephalitis had seizures, which manifested in three types: FAS (40%), FIAS (20%), and GTCS (100%), and 40% developed SE. The seizure semiology of the three cases with focal seizures varied, indicating different origins, which was consistent with the fact that anti-NMDAR encephalitis was diffuse encephalitis affecting extensive cortico-subcortical regions compared to classical limbic encephalitis (3, 31). These seizures showed high frequency and often developed SE in the second phase (psychotic and epileptic phase) of anti-NMDAR encephalitis whose clinical course progressed through five phases (32) and could be controlled when the disease progressed to subsequent stages. Previous studies (3, 5, 13, 29–31) showed that the incidence rate of seizures in anti-NMDAR encephalitis was ~70% (57–82%) and usually seen in the early phase, with various seizure types, more generalized seizures (53%) than focal seizures (17%), and often developed SE, which was consistent with our study. In a more recent study, 57% (43/75) of the patients with anti-NMDAR encephalitis developed seizures of the following types: tonic–clonic seizures (79%), focal seizures (74%) without impaired awareness (55%) or with impaired awareness (42%), and status epilepticus (35%). All of these 43 patients were seizure free after a median follow-up of 31 months (5). In another case, a series of 109 patients, 88 (81%) developed seizures, and all of them were seizure free at the 2 year follow-up (30). These and our study indicated that, in most patients, the seizures resolved after the encephalitis subsided.

The EEG is almost always (80–100%) abnormal in patients with anti-NMDAR encephalitis, which is non-specific and typically shows slowing or disappearing of background activities, focal or generalized slow waves, or interictal epileptic discharge (2, 3, 31, 33–35). The characteristic of EEG is extreme delta brush (EDB), which was first proposed by Schmitt et al. (36). The incidence of EDB in anti-NMDAR encephalitis was 5–33%, which was common in severe cases and associated with prolonged hospitalization, intensive care unit (ICU) admission, and poor prognosis (31, 33, 34, 36). Its pathophysiological mechanisms are still unknown and are thought to arise from disruption of glutamatergic neurotransmission, resulting in deafferentation and slow thalamocortical oscillations (31, 37). Gitiaux et al. (37) and other recent studies (31, 33, 34) suggested that the extent of EEG abnormalities correlated with clinical severity of anti-NMDAR encephalitis because the extent of slow waves in EEG was associated with the size of cortical regions affected. Focal abnormalities (slowing) on EEG were infrequent (13–67%) and distributed mainly in temporal or frontotemporal regions (31, 33, 34). Although clinical seizures are common (65.9%), electrographic evidence of epileptic activity is uncommon (15%), which potentially reflects a deeply located or highly localized epileptogenic zone (31). In our study, the abnormality rate of EEG was 100%, most of which showed diffuse or focal slow waves in background activities, and only 42.8% had interictal epileptic discharge, which was consistent with previous reports (2, 31, 33, 34). No patient showed extreme delta brush, which may be due to mild severity of all the cases in our group. However, two cases (2/7) showed delta activity or rhythm in the frontotemporal region, which was relieved as the patient's condition improved, indicating that this EEG pattern may be a specific feature of anti-NMDAR encephalitis. In addition, only 42.8% had abnormalities in brain MRI, which was consistent with previous studies showing that the MRI abnormality rate was low (only 30–40%) (31, 38). However, the abnormality rate of EEG was high and positively correlated with severity of the disease, further suggesting that EEG was more helpful than imaging in early diagnosis and outcome prediction of anti-NMDAR encephalitis.

In our study, the five patients with seizures were treated with corticosteroids combined with IVIG and AEDs, and all of them improved significantly and were seizure free after immunotherapy, suggesting good effect of immunotherapy. Previous studies showed that 75–81% of patients with anti-NMDAR encephalitis responded to immunotherapy (13). A recent study mentioned above showed that of the 43 patients with anti-NMDAR encephalitis who developed seizures, 39 (91%) survived the disease, and all were free of seizures after a median follow-up of 31 months. Seizure freedom was due to immunotherapy in 47% of patients and AEDs in 16%; for the rest of the patients, the cause of seizure freedom was unclear (combined effects of immunotherapy and AEDs, or spontaneous improvement). VPA, LEV, and CBZ were similarly effective, although CBZ was associated with fewer side effects (5, 29); however, sodium-channel blockers were recommended as first choice in some other studies (3, 5, 26, 39). In our study, the AED used in two cases was LEV; one was treated with LEV and TPM, one with VPA, and one with CBZ, which could not tell the preferable antiepileptic drug for the seizures in anti-NMDAR encephalitis. Several studies revealed that the seizures resolved after the encephalitis subsided and supported a gradual removal of antiepileptic therapy during the process of recovery (29, 30, 40). As reported, the recurrence rate was 12%, and the risk of developing chronic epilepsy was low (5, 13, 29). However, our study lacked sufficient statistical data due to short follow-up period. We need to continue to expand the number of cases and prolong follow-up period to further clarify the risk of developing chronic epilepsy and the recurrence rate of the disease.

### The Clinical and EEG Features of Seizures in Anti-GABA_B_R Encephalitis

In our study, both patients with anti-GABA_B_R encephalitis had seizures, which manifested in two types: GTCS and MTLE-like seizure, with status epilepticus; AEDs were ineffective, but immunotherapy was, which was in line with previous studies (2, 5, 6, 8, 13, 41–43). Previous studies have verified that severe and refractory seizures are the main features of anti-GABA_B_R encephalitis, as well as the initial and core symptoms, with incidence rate 90–100%. The common seizure types included focal seizure, FBTCS, and GTCS, with high frequency, and could rapidly progress to status epilepticus, and AEDs were ineffective (2, 5, 6, 8, 13, 41–43). There was correlation between seizures and disease activity (44). A recent study showed that drug-resistant seizures were the most frequent first symptom in 77% of the patients with anti-GABA_B_R encephalitis (45). The seizure types included focal (29%), FBTCS (24%), and GTCS (47%). In case of focal seizures, the symptoms suggested a medial temporal origin. The course of the disease were classified into three phases: (i) isolated seizure phase, (ii) encephalitic phase, and (iii) recovery phase. During the isolated seizure phase, no cognitive or mental impairment was observed, which may delay early diagnosis and treatment (3, 45).

According to previous studies, EEG showed diffuse or focal slow waves in background activities or interictal epileptic discharges in the temporal lobe; ictal EEG suggested medial temporal lobe origin (8, 42, 43, 45, 46). A recent report revealed that the distribution of slow wave activities reflected disease severity and may be related to disease recurrence (42). In our study, both patients had abnormalities in EEG; one showed focal slow waves in the left temporal region, and the other showed interictal epileptic discharges. One case had a subclinical electrographic seizure originating from the left temporal region, and EEG recorded 10 days after immunotherapy showed significant improvement in background activities, indicating that EEG was a good indicator of disease severity and effect of therapy, which was consistent with previous studies (8, 42, 43, 45, 46).

Immunotherapy should be started as soon as possible once the disease is diagnosed. Previous studies showed that 60% of patients with anti-GABA_B_R encephalitis responded to immunotherapy, and the risk of developing chronic epilepsy was 29% (13). A recent report showed that, compared with other types of AE such as anti-NMDAR encephalitis or LGI1 AE, the rate of seizure remission in anti-GABA_B_R encephalitis after 2 years follow-up was the lowest (55%), indicating the necessity of long-term AED treatment (8). Similarly, another study also suggested that patients with anti-GABA_B_R encephalitis had a higher risk of developing persistent seizures than anti-NMDAR encephalitis, but the mechanisms of which were still unknown (6). Both cases in our study showed good response to immunotherapy; the seizure was controlled after immunotherapy and AED treatment. One patient died of SCLC at 6 months after discharge, the other who achieved clinical remission and was seizure free discontinued oral corticosteroids and AED at 6 months. We need expand the sample number and prolong follow-up period to further clarify the long-term prognosis of this disease.

In summary, most patients with NSAb-associated AE have epileptic seizures, but the seizure semiology, clinical and EEG features, treatment, and prognosis of these seizures are different between different types of AE. LGI1 AE usually affected two locations in the brain: one was the limbic system, of which the seizure type at this time was MTLE-like seizure; the other was the basal ganglia, of which the seizure type was FBDS. Anti-NMDAR encephalitis had various seizure types, with more generalized than focal seizures, which often developed status epilepticus. The specific characteristic of EEG was extreme delta brush (seen in severe cases); delta activity or rhythm in the frontotemporal region was also helpful for diagnosis. Anti-GABA_B_R encephalitis was characterized by severe and refractory seizures, as well as initial and core symptoms, mainly GTCS and MTLE-like seizure, which could rapidly progress to status epilepticus, and AEDs were ineffective. Most seizures in NSAb-associated AE showed good response to immunotherapy, and AEDs should be considered as an add-on symptomatic treatment.

## Data Availability Statement

The raw data supporting the conclusions of this article will be made available by the authors, without undue reservation, to any qualified researcher.

## Ethics Statement

The studies involving human participants were reviewed and approved by the Medical Ethics Committee of the First Affiliated Hospital of Dalian Medical University, DaLian, China. The patients/participants provided their written informed consent to participate in this study.

## Author Contributions

YiW contributed to the study design and manuscript writing. YaW and YY contributed equally in clinical data collection, analysis, and manuscript writing. YH contributed to EEG analysis. YL contributed to imaging data analysis and figure preparation. FS revised and improved the manuscript. All authors read and approved the final manuscript.

## Conflict of Interest

The authors declare that the research was conducted in the absence of any commercial or financial relationships that could be construed as a potential conflict of interest.
